# Comparative analysis of skin transcriptome reveals differences of cashmere fineness in different body parts of Inner Mongolia cashmere goats

**DOI:** 10.5713/ab.25.0119

**Published:** 2025-07-11

**Authors:** Bohan Zhou, Haijiao Xi, Yongsheng Yu, Jinquan Li, Rui Su, Qi Lv, Yanjun Zhang, Ruijun Wang, Zhiying Wang

**Affiliations:** 1 College of Animal Science, Inner Mongolia Agricultural University, Hohhot, China; 2 Key Laboratory of Goat and Sheep Genetics, Breeding and Reproduction in Inner Mongolia Autonomous Region, Hohhot, China; 3 Key Laboratory of Mutton Sheep and Goat Genetics and Breeding, Ministry of Agriculture, Hohhot, China

**Keywords:** Cashmere Fineness Traits, Different Body Parts, Inner Mongolia Cashmere Goat, Skin Transcriptome

## Abstract

**Objective:**

The growth and development of secondary hair follicles primarily determine the economic value of cashmere traits, significantly influencing the quality of cashmere fineness. Previous studies have concentrated on the periodic growth regulation of hair follicles in Inner Mongolia cashmere goats (IMCGs), identifying numerous candidate genes that influence cashmere traits. Research on the factors and regulatory mechanisms affecting cashmere fineness in different body parts is currently limited.

**Methods:**

The differences of cashmere fineness traits among different body parts or ages were determined by multiple comparison analysis testing in analysis of variance. RNA-seq and Gene Ontology (GO) & Kyoto Encyclopedia of Genes and Genomes (KEGG) pathway enrichment analyses were used to assess the differentially expressed genes (DEGs) across different body parts of IMCGs. The candidate genes were validated using quantitative real-time polymerase chain reaction techniques.

**Results:**

Ages and different body parts had significant effects on cashmere diameter of IMCGs (p<0.05). Cashmere diameter was coarsest in the abdomen, but finest in the neck and back. A total of 2,178 DEGs were specifically screened among four body parts based on cashmere diameter. GO and KEGG analyses showed that these DEGs were mainly enriched in signaling pathways related to hair growth, such as MAPK signaling pathway and extracellular matrix-receptor interaction. The expression of *MATN2* and *CA12* were consistent with the phenotype of cashmere fineness in different body parts.

**Conclusion:**

The differences of cashmere fineness among different body parts of IMCGs were investigated through transcriptome and phenotype analysis, which provide a basis for understanding molecular regulation of cashmere growth in cashmere goats. *MATN2* and *CA12* have been validated as regulatory genes influencing the heterogeneity of cashmere fineness in various parts of IMCGs.

## INTRODUCTION

The Inner Mongolia cashmere goats (IMCGs), an excellent local cashmere and meat breed formed through long-term natural and artificial breeding, are farmed to provide high-quality cashmere [1]. The cashmere fineness refers to the diameter of cashmere fibers, which is generally below 16 μm. The finer the cashmere fineness, the higher the price. The cashmere fiber of IMCGs is pure white, soft, strong silky feeling, great elasticity and pure cashmere content which earn good reputation of “Fiber Diamond” and “Soft Gold” [2,3]. The applications of cashmere span across various fields, including textiles, traditional handicrafts and high-end luxury goods which are significant to the income increase of herders and the economic development of the Inner Mongolia region.

The hair types of cashmere goats belong to heterogeneous structures, with two different types of hair follicle structures including primary hair follicles (PHFs) and secondary hair follicles (SHFs) [4]. The development and growth of cashmere produced by SHF has a certain regularity, starting from the back, followed by the neck and body side, and finally the abdomen [5]. The PHFs growing wool are long and thick with large hair bulbs. The SHFs growing cashmere are short and fine with small hair bulbs. The growth pattern of wool and cashmere show strong seasonal variations mediated by circadian photoperiodicity. The hair cycle is also divided into three major stages, including anagen (growth phase), catagen (regression phase) and telogen (resting phase) [6,7]. There are some significant differences in cashmere of different parts of IMCGs, similar to the heterogeneity of hair of horses [8], pigs [9], humans [10] and other species from the phenotypic perspective.

RNA-sequencing (RNA-seq) is a high-throughput and high-sensitivity sequencing technology used to conduct differentially expressed genes (DEGs) studies at the whole genome level, providing more comprehensive and accurate RNA molecules [11]. Several RNA-seq studies have examined the molecular mechanisms of cashmere and identified DEGs between different cashmere types in cashmere goats. For example, fibroblast growth factor receptor 1 (*FGFR1*) and fibroblast growth factor 5 (*FGF5*), as important candidate genes in cell proliferation, activate MAPK and PI3K signaling pathways that play vital roles in morphological development of hair follicles from the perspective of cycle [12,13]. Matrilin 2 (*MATN2*) and tubulin polymerization promoting protein family member 3 (*TPPP3*) were significant DEGs between coarse cashmere and fine cashmere using RNA-seq and quantitative real-time polymerase chain reaction (qPCR) validation [14]. However, the molecular mechanisms responsible for cashmere fineness in different body parts are unknown. In this study, we investigated the significant differences in cashmere fineness among four body parts (neck, body side, back and abdomen) of IMCGs using analysis of variance. Moreover, the DEGs were identified and validated by transcriptomic data from the skin tissues of different body parts using transcriptomics analysis that may further discover the regulation of genes involved in the molecular mechanism of heterogeneity in cashmere.

## MATERIALS AND METHODS

### Animals and sample collection

Total 160 samples were taken from four parts (neck, body side, back, and abdomen) of 40 IMCGs ewes aged 2 and 5 in Inner Mongolia Jinlai Animal Husbandry Technology. Firstly, separate the cashmere and wool of the sample, then clean the grease and sediment of the cashmere with petroleum ether, further dry the surface petroleum ether with filter paper, and finally measure the cashmere diameter (CD) and diameter variable coefficient (DVC) using OFDA2000 (BSC Electronics).

The effects of age and different body parts on cashmere fineness of IMCGs were determined using SAS software (v9.2) for variance analysis and multiple comparisons. The general linear model (GLM) is a widely utilized statistical learning framework that achieves superior data fitting and enables systematic investigation of interaction effects. The GLM was as follows:


(1) $$y={age}_{i}+body\;{part}_{j}+{\left(age\times body\;part\right)}_{ij}+e_{ij}$$

Where *y* is the vector of observations for each trait (CD or DVC), *age_i_* is the vector of *i* years old (2 or 5), *body part_j_* is the vector of *j* each body part (body side or abdomen or back or neck), (*age*×*body part*)*_ij_* is the interaction vector between the *i* years old and *j* body part, *e_ij_* is the vector of residual effects.

The animal experiments were approved by the Animal Welfare and Ethics Committee of Inner Mongolia Agricultural University (IACUC APPROVAL NUMBER: NND2025099). The skin tissue of four parts from three 5 years-old IMCGs ewes were selected as experimental samples for RNA sequencing. We divided the samples into neck (n = 3), body side (n = 3), back (n = 3), and abdomen (n = 3). The number and name of samples from different body parts of IMCGs were listed in Supplement 1. The samples were delivered in dry ice and stored at −80°C until RNA-seq.

### RNA extraction and quality assessment

Total RNA of skin tissue was extracted using TRIzol and RNA extraction kit (Invitrogen). Agarose gel electrophoresis was used to analyze the degree of RNA degradation and whether there were contaminants. NanoDrop 2000 (Thermo Fisher Scientific) was used to detect the purity of RNA and OD260/280 ratio, and 2100 bioanalyzer (Agilent) was used to accurately detect the integrity of RNA. High-quality RNA samples were used to construct the sequencing library.

### Library construction and RNA-Seq

The cDNA library was constructed and sequenced using NEBNext Ultra RNA Library Prep Kit for Illumina. The mRNA with polyA tails was enriched using Oligo (dT) beads and fragmented into small pieces [15]. The first strand of cDNA was synthesized in the M-MuLV reverse transcriptase system, using fragmented mRNA as a template and random oligonucleotides as primers. The second strand of cDNA was synthesized in the DNA polymerase I system, using dNTPs as substrates. The end repair, A-tail addition and ligation of sequencing adapters of purified double-stranded cDNA were performed according to the manufacturer’s instructions. The cDNA fragments (250 bp to 300 bp) were selected followed by PCR amplification. Paired-end libraries were sequenced using Illumina Novaseq 6000 sequencing (150 bp×2; Novogene) after quantification using Qubit2.0 Fluorometer [16].

### Reads quality control and sequence alignment analysis

The raw reads obtained from sequencing contained a small number of reads with sequencing adapters or low quality. Therefore, the clean reads used in the subsequent analysis were obtained after the raw reads filtering, sequencing error rate checking and GC content distribution checking. The clean reads were separately aligned to the Capra hircus (goat) genome (ARS1) including localization of exon region, intron region and intergene region of the genome using hisat2 (v2.0.5) software [17]. The new assembled transcripts were merged using StringTie (v1.3.3) software.

### Differentially expressed genes analysis

To identify the DEGs between different experimental groups, Fragments Per Kilobase Million (FPKM) was used to calculate the expression level of each gene. The R package DESeq2 (v1.46.0) was used to detect DEGs between two comparison groups [18]. We selected significant DEGs based on *P*adj<0.05 and |log2(FoldChange)|>0.

### Gene Ontology enrichment and Kyoto Encyclopedia of Genes and Genomes analysis

To determine the biological significance of the DEGs, Gene Ontology (GO) annotation and Kyoto Encyclopedia of Genes and Genomes (KEGG) pathway gene annotation were carried out functional enrichment analysis of candidate genes using clusterProfiler (v3.4.4). The GO enrichment analysis can be divided into biological process (BP), cellular component (CC) and molecular function (MF) [19]. The adjusted p-value<0.05 was used to determine significantly enriched pathways associated with the DEGs.

### Quantitative real-time polymerase chain reaction validation

qPCR to confirm the repeatability of DEGs from RNA-Seq. Aquaporin 5 (*AQP5*) (*P*adj = 0.02, log2FoldChange = 4.58), Matrilin 2 (*MATN2*) (*P*adj = 0.04, log2FoldChange = 2.96), Carbonic Anhydrase 12 (*CA12*) (*P*adj = 0.01, log2FoldChange = 3.26) were randomly selected as representative significant DEGs for qPCR validation. Gene expression was examined for *AQP5*, *MATN2*, *CA12* in different body parts of IMCGs using TB Green Premix Ex Taq II (TaKaRa). The qPCR reaction was performed with an initial denaturation at 95°C for 2 min, followed by 40 cycles of 98°C for 30 s, 60°C for 30 s, and 72°C for 30 s. The qRCR primers for target genes were designed using Primer Premier (v5.0) [20]. The primer sequences, gene length and amplification temperatures of target genes were listed in Supplement 2. The expressions of relative genes were calculated by using the 2−ΔΔCt method and the β-actin was selected as housekeeping gene for normalization. The qPCR experiments were performed with three technical replicates.

### Statistical analysis

A 2-tailed unpaired t-test was performed for the relative expression level of DEGs using SAS software (v9.2). The results were presented as the mean±standard deviation (SD) and the significant difference was set at p<0.05.

## RESULTS

### Comparison of cashmere fineness of female Inner Mongolia cashmere goats at different body parts or ages

The average 159 (1 failure) CD of IMCGs is 14.68 μm, with SD of 0.6758 μm. The average 160 DVC of IMCGs is 23.09% with SD of 2.4956% (Table 1). Ages had significant effects on CD and DVC (p<0.05), but different body parts had only significant effects on CD (p<0.05). The interaction between ages and different body parts had no significant effects on CD and DVC of IMCGs (p>0.05) (Supplement 3).

**Figure 1:**
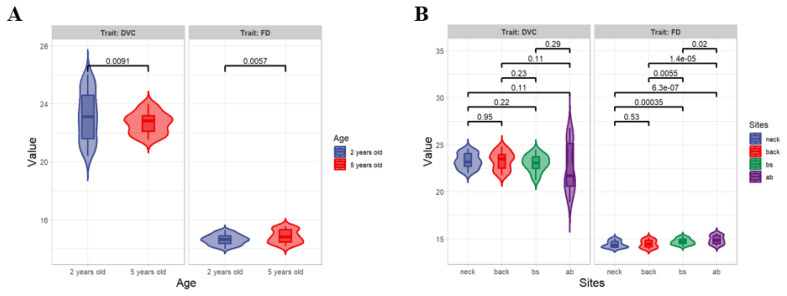
Violin Diagram showing comparison analysis results of diameter variable coefficient (DVC) and fiber diameter (FD) of IMCGs. (A) Comparison analysis of DVC and FD at the different ages. (B) Comparison analysis of DVC and FD at the different body parts. IMCGs, Inner Mongolia cashmere goats.

The CD and DVC of IMCGs age 5 years old was significantly higher than that of IMCGs age 2 years old (p<0.05) (Figure 1A; Table 2). The CD of body side and abdomen of IMCGs was significantly higher that of neck and back (p< 0.05). The CD of abdomen of IMCGs was significantly higher that of other body parts (p<0.05). However, there was no significant difference on the DVC of different body parts of IMCGs (p>0.05) (Figure 1B; Table 2).

### RNA sequencing and quality control

Raw reads from four different parts (neck, body side, back and abdomen) of IMCGs were collected using RNA sequencing. A total of 563,224,190 raw reads with an average of 46,935,349 reads and 546,215,960 clean reads with an average of 45,517,996 reads were obtained after filtering out low-quality, spliced and duplicate reads. The quality control results showed that Q20 and Q30 of the samples were over 97.37% and 92.75%, the compare rates of error were about 0.02 or 0.03 indicating the sequencing results were reliable and could be used for subsequent analysis (Supplement 4).

### Identification of differentially expressed genes

**Figure 2:**
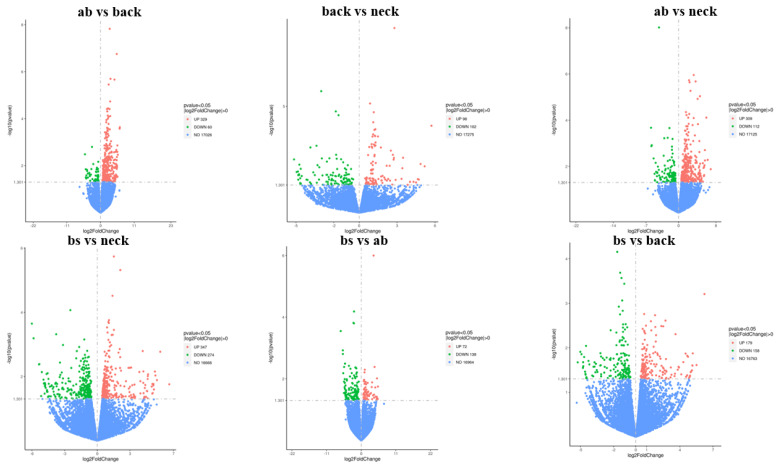
Volcano plots showing upregulated and downregulated DEGs between different comparisons. DEG, differentially expressed gene.

The log_10_ (FPKM+1) values of repeated samples between different parts were generally same, indicating that the gene expression levels of samples from different parts of IMCGs were similar approximately (Supplement 5). DEGs were filtered with p-value<0.05 and |log_2_(fold change)|>0. 389 DEGs were identified, with 329 upregulated and 60 downregulated genes in the abdomen (ab) vs back comparison. 200 DEGs were identified, with 98 upregulated and 102 downregulated genes in the back vs neck comparison. 421 DEGs were identified, with 309 upregulated and 112 downregulated genes in the abdomen (ab) vs neck comparison. 621 DEGs were identified, with 347 upregulated and 274 downregulated genes in the body side(bs) vs neck comparison. 210 DEGs were identified, with 72 upregulated and 138 downregulated genes in the body side (bs) vs abdomen (ab) comparison. 337 DEGs were identified, with 179 upregulated and 158 downregulated genes in the body side (bs) vs back comparison. The upregulated genes were more than downregulated genes in the four comparisons, except for the two comparisons (back vs neck, bs vs ab) (Figure 2).

### Analysis of co-expressed differentially expressed genes

**Figure 3:**
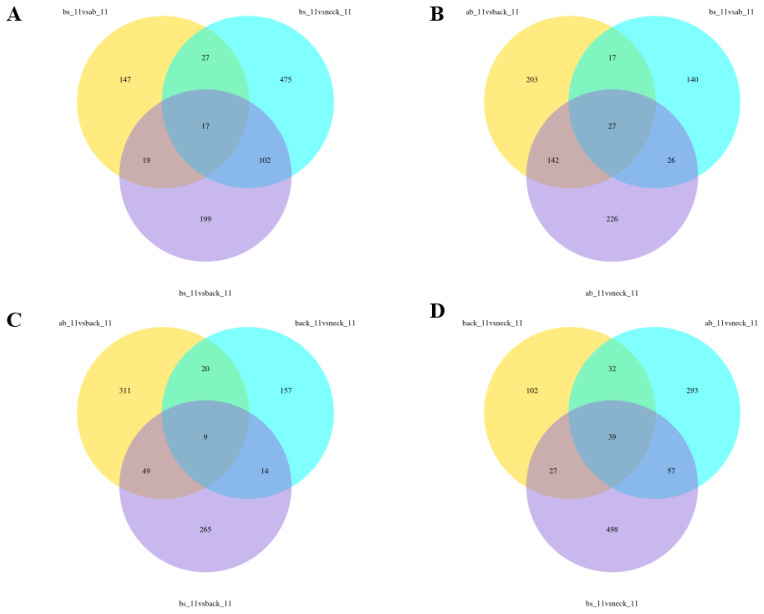
Venn diagram of overlapping co-DEGs among different comparisons. (A) co-DEGs among bs vs other body parts, (B) co-DEGs among ab vs other body parts, (C) co-DEGs among back vs other body sites, (D) co-DEGs among neck vs other body parts. DEG, differentially expressed gene.

The co-expressed differentially expressed genes (co-DEGs) between a certain body part and three other parts were shown using Venn diagram analysis. 17 co-DEGs were identified in the three comparisons (bs vs ab, bs vs neck, bs vs back). 27 co-DEGs were identified in the three comparisons (ab vs back, ab vs bs, ab vs neck). 9 co-DEGs were identified in the three comparisons (back vs ab, back vs neck, back vs bs). 39 co-DEGs were identified in the three comparisons (neck vs back, neck vs ab, neck vs bs) (Figures 3A–3D). The 8 co-DEGs including Keratin 14 (*KRT14*), Carbonic Anhydrase 12 (*CA12*), ras related dexamethasone induced 1 (*RASD1*), FosB Proto-Oncogene, AP-1 Transcription Factor Subunit (*FOSB*), Corneodesmosin (*CDSN*), Aquaporin 5 (*AQP5*), Wnt Family Member 4 (*WNT4*), Matrilin 2 (*MATN2*) among different comparisons showed a high degree of significance (p<0.01).

### Functional annotation of differentially expressed genes

**Figure 4:**
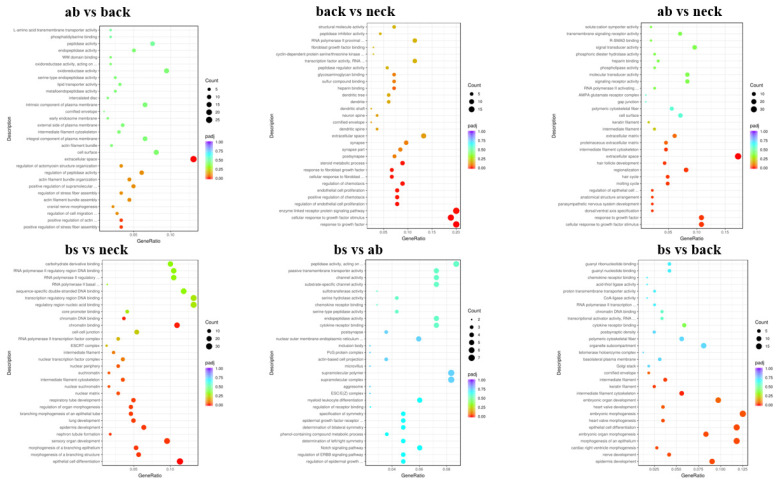
GO enrichment analysis of DEGs. GO, Gene Ontology; DEG, differentially expressed gene.

**Figure 5:**
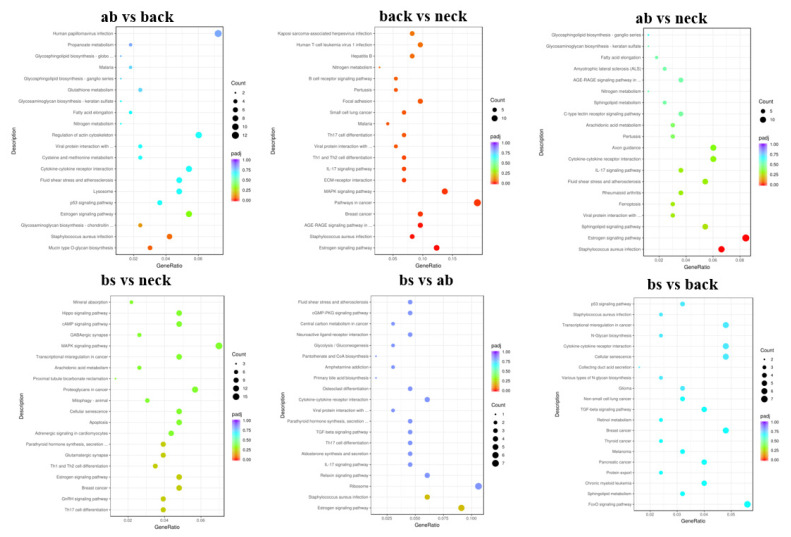
KEGG pathway enrichment analysis of DEGs. KEGG, Kyoto Encyclopedia of Genes and Genomes; DEG, differentially expressed gene.

To further elucidate the functions of the DEGs, GO and KEGG analysis were performed. In the two comparisons (ab vs back, ab vs neck), the DEGs were mainly enriched in extracellular space (GO:0005615). In the two comparisons (neck vs ab, neck vs back), the DEGs were mainly enriched in response to growth factor (GO:0070848) and cellular response to growth factor stimulus (GO:0071364). In the two comparisons (bs vs back, bs vs neck), the DEGs were mainly enriched in epithelial cell differentiation (GO:0030855). Besides, the DEGs were mainly enriched in positive regulation of stress fiber assembly (GO:0051496) and positive regulation of actin filament bundle assembly (GO:0032233) in the ab vs back comparison. The DEGs were mainly enriched in enzyme-linked receptor protein signaling pathway (GO:0007167) in the back vs neck comparison. The DEGs were mainly enriched in embryonic morphogenesis (GO:0048598) and morphogenesis of an epithelium (GO:0002009) in the bs vs back comparison. The DEGs were mainly enriched in sensory organ development (GO:0007423) and chromatin binding (GO:0003682) in the bs vs neck comparison (Figure 4). Besides, KEGG analysis showed that DEGs among six different comparisons were significantly enriched in 24 pathways (p<0.05). *DUSP1*, *TGFBR2*, and *FOS* genes were significantly enriched in the mitogen-activated protein kinase (MAPK) signaling pathway, while KRT family genes (*KRT25*, *KRT27*, *KRT28*, *KRT19*) and *MMP9* gene showed significant enrichment in the estrogen signaling pathway (Figure 5).

### Validation of differentially expressed genes using quantitative real-time polymerase chain reaction

**Figure 6:**
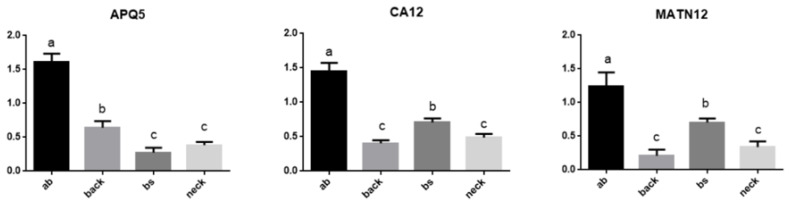
Relative expression of *AQP5*, *MATN2*, and *CA12* at different body parts. Relative expression of *AQP5*, *MATN2*, and *CA12* at different body parts. Different letters (a−c) meant there was significant difference among groups (p<0.05)

To identify candidate genes associated with cashmere fineness in different body parts of IMCGs, three genes (*AQP5, MATN2, CA12*) selected from the most significant pathways, such as MAPK signaling pathway, extracellular matrix (ECM) receptor interaction and Estrogen signaling pathway were measured using qPCR. The results showed that all genes showed a similar expression pattern in both RNA-seq and qPCR, indicating that the RNA-seq data were reliable and accurate (Figure 6; Supplement 6). The expression levels of these genes in abdomen were higher than in the other body parts (p<0.05). Moreover, the expression levels of *MATN2* and *CA12* were consistent with the phenotype of cashmere fineness in different body parts.

## DISCUSSION

Cashmere is one of the most important economic traits of cashmere goats, of which fineness is the key indicators to determine the quality of cashmere [21,22]. The finer the cashmere, the better the hand handle and warm-keeping ability. The cashmere of IMCGs is of premium quality (with CD<15 μm) and occupies the important position in high-end luxury market [23,24]. In this study, Age had the significant impacts on the cashmere fineness traits of IMCGs such as CD DVC. The CD of 5-year-old individuals were significantly higher than those of 2-year-old individuals. On the contrary, DVC of 5-year-old individuals were significantly smaller than that of 2-year-old individuals.

Previous studies showed the cashmere became coarser and the DVC gradually decreased in goats and sheep such as Liaoning cashmere goats [25], Alax White cashmere goats [26], Altay sheep [27] with increasing age. Besides, the different parts only had the significant impact on CD of IMCGs, with the finest cashmere (14.50 μm) on the back or neck, moderate cashmere (14.72 μm) on the body side and the coarsest cashmere (15.00 μm) on the abdomen. The variation in cashmere across different body parts of Liaoning cashmere goats was observed to range from the coarsest to the finest, with the following order of CD: back>hip>shoulder>body side>abdomen [25]. The finest cashmere were located on the abdomen, measuring 15.12 μm, while the coarsest cashmere were found on the back, measuring 15.96 μm. For Shaanbei white cashmere goats, the hip’s cashmere was the coarsest, reaching 16.50 μm and the abdomen’s cashmere was the finest, reaching 15.72 μm [28]. Alxa cashmere goats were similar to IMCGs, with relatively fine cashmere on the back and neck, measuring 15.7 μm and 16.3 μm respectively, while relatively coarse cashmere on the body side and abdomen, measuring 16.5 μm and 17.1 μm respectively [29]. Therefore, there is a certain regularity in the variation of cashmere fineness among body parts of cashmere goats, but the pattern also varies among different varieties. Compared with other cashmere goats, IMCGs have finer and better-quality cashmere among different body parts [30]. In production practice, it is necessary to implement the collection of cashmere in different body parts to further achieve high-quality and cost-effective sales in the cashmere market.

The transcriptome is the sum of all RNAs that can be transcribed from a living cell at a specific time and environment [31]. The differential gene expression (DGE) analysis of RNA-seq is a bioinformatics analysis method that aims to compare the differences in transcriptome sequencing data under different samples or conditions to reveal changes in gene expression and regulatory mechanisms [32,33]. In this study, RNA-seq analysis was performed on 12 samples from four body parts of IMCGs and the candidate genes including *WNT4, FOSB*, *KRT14* may cause heterogeneity of cashmere among different body parts.

The WNT/β - Catenin signaling pathway plays an important role in regulating hair follicle morphology [34]. It is speculated that DEGs in the WNT signal transduction pathways such as Wnt Family Member 3 (*WNT3*) and Wnt Family Member 10A (*WNT10A*) may affect the embryonic initiation and growth development of hair follicles in IMCGs [35]. The Keratin-associated proteins (KAP) are components of cashmere and many variants of the KAP genes (KRTAPs) are related to the cashmere traits. It is suggested that KRTAP28-1 gene has five variants (A–E), of which variant A resulted in the reduction of mean CD [36]. Moreover, the *KAP15-1* gene was expressed in the SHFs of Longdong cashmere goats, but not in other organs such as the heart, liver, spleen and lungs by RT-PCR validation [37]. The Keratin 2 (KRT2) gene was also associated with CD using genome-wide association study of cashmere traits [38]. It was found that *FOSB*, *KRT85*, *KRT33A* might participate in the differentiation and development of hair follicles using transcriptome analysis of coarse cashmere type and fine cashmere type Tibetan cashmere goats [39].

In this study, DEGs in the comparisons (back vs neck) were mainly enriched in the MAPK signaling pathway. Previous studies have indicated that MAPK signaling pathway is critical for hair follicle stem cell (HFSC) proliferation, apoptosis and differentiation [40,41]. The MAPK signaling pathway plays an important role in the three stages of hair follicle development [42]. It promoted hair formation by stimulating the proliferation of HFSCs especially during the growth phase. In addition, the MAPK signaling pathway interacted with transforming growth factor-β/bone morphogenetic protein (TGF-β/BMP) to jointly regulate the growth and development of hair follicles [43]. The MAPK signaling pathway can also affect the quality and characteristics of hair. It can affect the thickness and strength of cashmere through regulating the differentiation of HFSC [44].

In this study, there were significant differences in the expression of *AQP5*, *MATN2*, and *CA12* genes in four parts (abdomen, back, body side and neck) of IMCGs. Several aquaporins (AQPs) are expressed in mammalian skin. For example, *AQP5* was mainly expressed on the cell membrane of keratinocytes in the granular layer, playing an important role in maintaining water balance between skin cells [45]. ECM has impact on tissue differentiation, maintenance and remodeling during development and regeneration of skin [46]. *MATN2*, the components of ECM, may provide structural support of skin cells by participating in regulating intercellular interactions and signal transduction [47]. In addition, there are the expressions of other DEGs among different body parts of the skin or hair follicles in some species. The mRNA of *TGF-β2* in scrotum and axillary was significantly higher than those in abdomen and back of young Yak. The mRNA of *HIF-1α* in the abdomen was significantly higher than those in the other three sites (neck, back and chest) [48]. TGF-βR1and p38 MAPK may be related to the growth and development of sheep hair. The expression level of TGF-βR1 was highest in the ear and lowest in the back. However, the expression level of p38 MAPK was highest in the back and lowest in the groin [49]. R-Spondin 2 (*RSPO2*) is crucial for the development of limbs, lungs and hair follicles [50,51]. *β-catenin* is an important adhesion molecule in epithelial tissue, located downstream of the Wnt pathway. It affects the hair cycle by inducing the occurrence of hair follicle morphology and the proliferation of HFSC. qPCR showed that the expression of *RSPO2* in the back and the leg were 7.633 folds and 5.602 folds respectively in comparison with that in the ear. The expression of *β-catenin* in the back and the leg were 3.689 folds and 2.067 folds respectively in comparison with that in the ear [52].

## CONCLUSION

There were the significant differences in CD among four different body parts (abdomen, body side, back, neck) of IMCGs. The CD of the abdomen was significantly coarser than the other three body parts, with the pattern of size change was abdomen>body side>neck>back. However, there were no significant differences in the DVC among four different body parts of IMCGs. DEGs such as *FOSB*, *KRT14*, *CA12*, *WNT4*, *AQP5*, *CAPN5*, *ASAP1* and *MATN2* were found to be enriched in some pathways about hair follicle growth and development using RNA-seq. *MATN2*, *CA12* have been validated as regulatory genes for the heterogeneity of cashmere fineness in different parts of IMCGs. These results provided some important genes regulating cashmere fineness using joint analysis of phenotypes and transcriptomes which laid a certain theoretical basis for molecular breeding of cashmere goats.

## Figures and Tables

**Table 1:** Basic descriptive statistics of cashmere fineness traits in IMCGs

Traits	Number	Mean	SD	CV (%)	Max	Min
FD (μm)	159	14.68	0.6758	4.60	16.73	13.40
DVC (%)	160	23.09	2.4956	10.81	32.8	6.90

IMCGs, Inner Mongolia cashmere goats; SD, standard deviation; CV, coefficient of variation; FD, fiber diameter; DVC, diameter variable coefficient.

**Table 2:** Comparison analysis results of FD and DVC of IMCGs

Factors	Levels	FD			DVC		
	
		Number	Mean±SD	p-value	Number	Mean±SD	p-value
Age	2 years old	116	14.62±0.66^b^	0.0057^**^	116	23.21±2.81^a^	0.0091^**^
	5 years old	43	14.85±0.69^a^		44	22.73±1.23^b^	
Body parts	Neck	39	14.50±0.56^c^	0.0008^**^	40	23.60±1.66^ns^	0.0747^ns^
	Back	40	14.50±0.64^c^		40	23.10±1.75^ns^	
	Body side	40	14.72±0.58^b^		40	22.88±1.65^ns^	
	Abdomen	40	15.00±0.76^a^		40	22.78±4.05^ns^	

a–c The different superscript letter indicates a significant difference (p<0.05); the same superscript letter indicates a non-significant difference (p>0.05).

** Indicates highly significant difference (p<0.01).

FD, fiber diameter; DVC, diameter variable coefficient; IMCGs, Inner Mongolia cashmere goats; SD, standard deviation.

## SUPPLEMENTARY MATERIAL

**Supplement 1.** Number and name of samples from different body parts of IMCGs. **Supplement 2.** Primer sequence information of DEGs.

**Supplement 3.** Variance analysis results of fiber diameter and diameter variable coefficient of IMCGs.

**Supplement 4.** Summary of sequencing data quality.

**Supplement 5.** Gene expression and correlation analysis among samples.

**Supplement 6.** Comparison analysis results of relative expression of candidate genes in different body parts.
