# Supplementary material for: Comparative analysis of skin transcriptome reveals differences of cashmere fineness in different body parts of Inner Mongolia cashmere goats
Source: Anim Biosci. 2025 Jul 11;38(12):2612–23. doi: 10.5713/ab.25.0119 (PMC12580752; doi:10.5713/ab.25.0119)
Supplement: Supplementary file 1 [file ab-25-0119-Supplementary-1,2.pdf]

Supplement 1. Number and name of samples from different body parts of IMCGs

	Body side	Abdomen	back	neck
Number and name	bs_1	ab_1	back_1	neck_1
	bs_2	ab_2	back_2	neck_2
	bs_3	ab_3	back_3	neck_3
Group name	bs_11	ab_11	back_11	neck_11

Supplement 2. Primer sequence information of DEGs

Gene name	Primer sequence (5'→3')	Product length（bp）	Tm（℃）
β-actin	F: GGCAGGTCATCACCATCGG<br>R: CGTGTTGGCGTAGAGGTCTTT	158	60
AQP5	F: CGGGCCGCCTGCCTGGTATA<br>R: GCCCGGCGGACGGACCATAT	138	60
MATN2	F: GGCTGCTTTCTGCTGCTCTTCG<br>R: CCGACGAAAGACGACGAGAAGC	310	58.6
CA12	F: TGGTGTTCTTGGCATCTGTATT<br>R: ACCACAAGAACCGTAGACATAA	126	56
