# Supplementary material for: Comparative analysis of skin transcriptome reveals differences of cashmere fineness in different body parts of Inner Mongolia cashmere goats
Source: Anim Biosci. 2025 Jul 11;38(12):2612–23. doi: 10.5713/ab.25.0119 (PMC12580752; doi:10.5713/ab.25.0119)
Supplement: Supplementary file 2 [file ab-25-0119-Supplementary-3.pdf]

Supplement 3. Variance analysis results of fiber diameter and diameter variable coefficient of IMCGs

Traits / Fixed Factors	DF	FD				DVC			
		SS	MS	*F*-value	*P*-value	SS	MS	*F*-value	*P*-value
Age	1	1.70	1.70	4.18	0.0057**	0.012	0.012	0.070	0.0091**
Body parts	3	6.91	2.30	5.65	0.0008**	0.066	0.022	0.140	0.0747^{ns}
Age*Body parts	3	1.96	0.65	1.61	0.1904^{ns}	0.893	0.298	1.900	0.1323^{ns}

Note: CD: cashmere diameter; DVC: diameter variable coefficient, Age*Body parts: interaction effects of age and body parts, **: indicates highly significant difference ($P < 0.01$); *: indicates significant difference ($P < 0.05$).
