# Supplementary material for: Comparative analysis of skin transcriptome reveals differences of cashmere fineness in different body parts of Inner Mongolia cashmere goats
Source: Anim Biosci. 2025 Jul 11;38(12):2612–23. doi: 10.5713/ab.25.0119 (PMC12580752; doi:10.5713/ab.25.0119)
Supplement: Supplementary file 3 [file ab-25-0119-Supplementary-4.pdf]

Supplement 4. Summary of sequencing data quality

sample	raw_reads	clean_reads	clean_bases	error_rate	Q20	Q30
ab_1	47417300	45781278	6.87G	0.03	97.57	93.18
ab_2	47068026	45344448	6.8G	0.03	97.39	92.77
ab_3	46734694	44680324	6.7G	0.02	98.04	94.67
back_1	47193470	46013042	6.9G	0.03	97.57	93.19
back_2	47309012	46179960	6.93G	0.03	97.49	93.09
back_3	46766880	45185544	6.78G	0.03	97.61	93.24
bs_1	49871180	48441760	7.27G	0.03	97.53	93.26
bs_2	44503730	43253132	6.49G	0.03	97.37	92.75
bs_3	45223434	43722862	6.56G	0.03	97.78	93.67
neck_1	47073744	46010080	6.9G	0.03	97.75	93.58
neck_2	47377934	46052576	6.91G	0.03	97.38	92.88
neck_3	46684786	45550954	6.83G	0.03	97.42	92.93
