# Supplementary figures and images for: Comparative analysis of skin transcriptome reveals differences of cashmere fineness in different body parts of Inner Mongolia cashmere goats
Source: Anim Biosci. 2025 Jul 11;38(12):2612–23. doi: 10.5713/ab.25.0119 (PMC12580752; doi:10.5713/ab.25.0119)

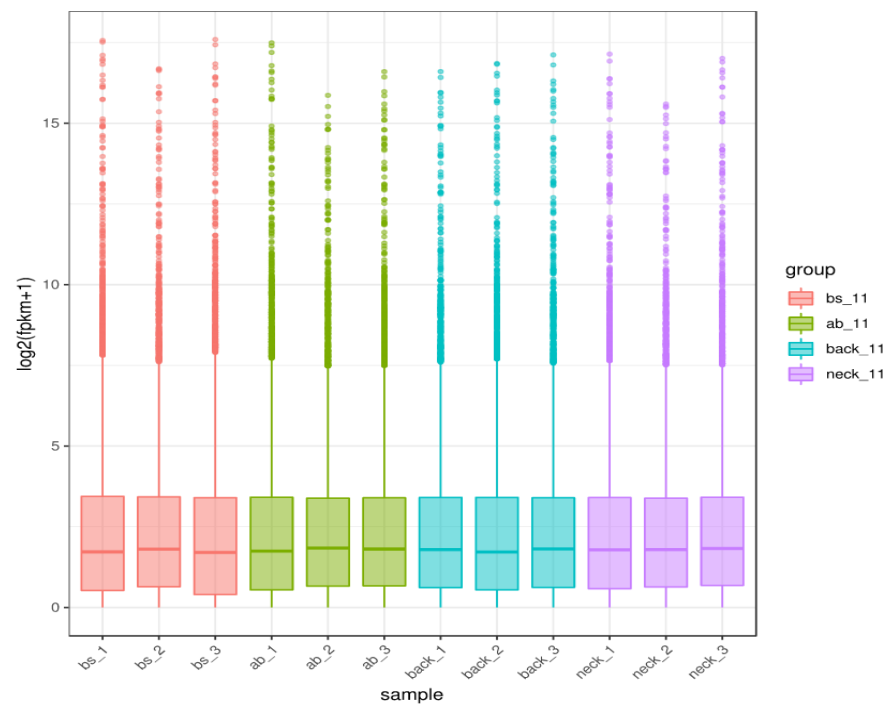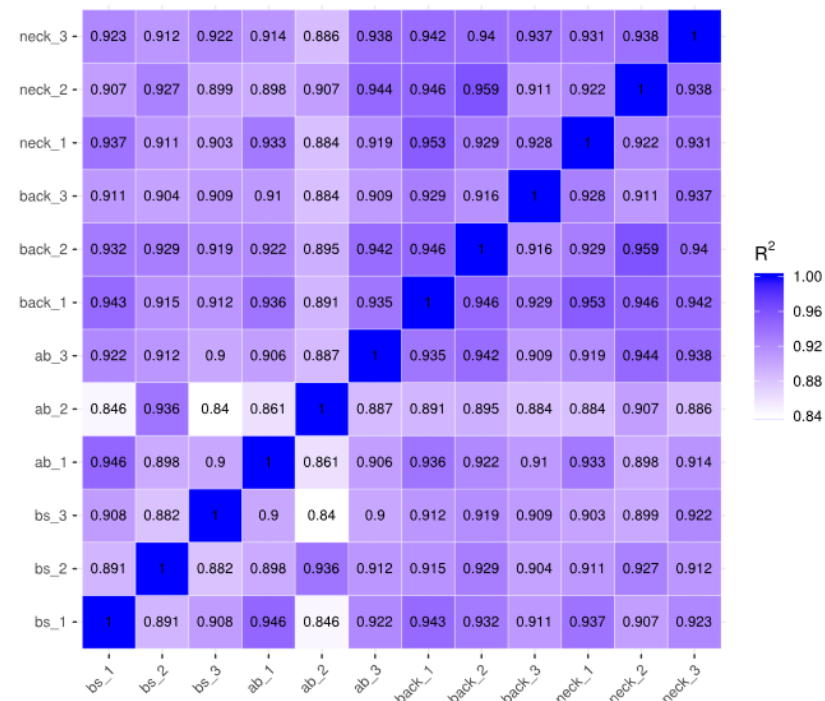

Supplement 5. Gene expression and correlation analysis among samples

Supplement: Supplementary file 4 [file ab-25-0119-Supplementary-5.pdf]
