# Supplementary material for: Comparative analysis of skin transcriptome reveals differences of cashmere fineness in different body parts of Inner Mongolia cashmere goats
Source: Anim Biosci. 2025 Jul 11;38(12):2612–23. doi: 10.5713/ab.25.0119 (PMC12580752; doi:10.5713/ab.25.0119)
Supplement: Supplementary file 5 [file ab-25-0119-Supplementary-6.pdf]

Supplement 6. Comparison analysis results of relative expression of candidate genes in different body parts

gene	Abdomen	back	Body side	neck
APQ5	1.61 ± 0.13^{a}	0.64 ± 0.1^{b}	0.27 ± 0.07^{c}	0.38 ± 0.05^{c}
CA12	1.45 ± 0.12^{a}	0.40 ± 0.04^{c}	0.71 ± 0.06^{b}	0.49 ± 0.05^{c}
MATN12	1.24 ± 0.21^{a}	0.21 ± 0.09^{c}	0.70 ± 0.06^{b}	0.34 ± 0.09^{c}
